# Comprehensive impact of Intermittent Hypoxia Training and Intermittent Fasting on metabolic and cognitive health in adults with obesity: an umbrella systematic review and meta-analysis

**DOI:** 10.3389/fnut.2025.1664600

**Published:** 2025-10-15

**Authors:** Jiajia Guo, Ning Zhang, Juan Chen, Xueyu Liu

**Affiliations:** Department of Nursing, Lianyungang Higher Vocational College of Traditional Chinese Medicine, Lianyungang, Jiangsu, China

**Keywords:** Intermittent Fasting, metabolism, cognition, obesity, Intermittent Hypoxia Training

## Abstract

**Background:**

Obesity has emerged as a global health crisis, posing significant challenges to metabolic function and cognitive health. It is associated with insulin resistance, elevated triglycerides, and reduced HDL cholesterol levels. Cognitive decline in obesity involves multifactorial pathways including neuroinflammation, vascular dysfunction, and blood–brain barrier disruption. Intermittent Hypoxia Training (IHT) and Intermittent Fasting (IF) have shown promise as non-pharmacological interventions for these obesity-related issues.

**Objectives:**

This systematic review and meta-analysis aim to evaluate the effects of IHT, IF, and their combination on metabolic markers (insulin sensitivity, lipid profiles, glucose levels) and specific cognitive domains (memory, executive function, attention) and cognitive function in obese adults.

**Methods:**

The analysis encompassed studies published from October 2014 onward, sourced from PubMed, Web of Science, Scopus, and other relevant databases. The inclusion criteria were randomized controlled trials and non-randomized comparative studies focusing on IHT, IF, or their combination. Study quality was assessed using the Newcastle-Ottawa Scale and the Cochrane risk bias tool. Data synthesis was performed using a random-effects model, with heterogeneity assessed via *I*^2^ statistics.

**Results:**

The review included 28 studies involving 2,134 participants, followed up for an average of 12 weeks. Among these, 15 were RCTs and 13 were observational studies. The participants had a mean age of 45 ± 12 years, with 60% being female. The combined IHT and IF intervention demonstrated superior benefits, with significant weight loss (mean reduction: 6.3 kg, 95% CI: −8.2 to −4.5 kg). Cognitive performance showed domain-specific improvements: memory (SMD = 0.60, 95% CI: 0.43–0.77) and attention (SMD = 0.57, 95% CI: 0.40–0.74), though with significant heterogeneity (*I*^2^ > 50%). Egger’s test indicated minimal publication bias (*p* = 0.18).

**Conclusion:**

Our meta-analysis reveals that IHT and IF may serve as promising non-drug strategies for improving metabolic and cognitive outcomes in adults with obesity. Given the short-term evidence and methodological heterogeneity, long-term studies are needed to confirm these findings.

## Highlights

- The meta-analysis confirms that Intermittent Hypoxia Training (IHT) and Intermittent Fasting (IF) significantly aid in weight loss, improve glucose metabolism, enhance lipid profiles, and boost cognitive function.
- The combination of IHT + IF is particularly effective, showing an average weight loss of 6.3 kg and substantial improvements in insulin sensitivity, cholesterol levels, and cognitive abilities.
- These interventions serve as viable, non-pharmacological alternatives to medication, especially for individuals who prefer lifestyle modifications or cannot tolerate medications.

## Background

Obesity has emerged as a major public health challenge in the 21st century, imposing a considerable burden on both population health and healthcare systems. Projections suggest that by 2025, globally, 18% of men and 21% of women will be affected by obesity (1). This epidemic not only escalates the risk of various physical ailments but also is associated with cognitive decline, thereby diminishing the quality of life (2). The underlying mechanisms involve neuroinflammation, vascular dysfunction, and blood–brain barrier disruption (3). Obesity is intricately linked to metabolic disorders. Insulin resistance (IR), which affects approximately 40% of individuals with obesity (4), compromises the body’s ability to efficiently absorb and utilize blood glucose, leading to hyperglycemia and potentially progressing to type 2 diabetes (T2D). Additionally, obesity is associated with dyslipidemia, characterized by elevated levels of low-density lipoprotein (LDL) cholesterol and triglycerides (TG), alongside reduced levels of high-density lipoprotein (HDL) cholesterol. These metabolic abnormalities, in conjunction with chronic low-grade inflammation fueled by elevated pro-inflammatory organokines (e.g., leptin, adiponectin) (4, 5), establish a detrimental feedback loop that accelerates disease progression and severely affects overall health (4–6).

Obesity also adversely affects brain function, impairing executive abilities, memory processes, and decision-making capacity. Individuals with obesity-related hypoxemia (e.g., from sleep apnea) are at a heightened risk of developing Alzheimer’s disease (AD) and other forms of dementia (3, 4). Beyond inflammation and oxidative stress, these cognitive impairments involve mitochondrial dysfunction and altered neurotrophic factor signaling (3).

Against this backdrop, two emerging strategies show considerable promise for obesity management: Intermittent Hypoxia Training (IHT) and Intermittent Fasting (IF). IHT involves exposing individuals to controlled, reduced oxygen levels (typically 10–15% O₂) that replicate high-altitude conditions. This method has been demonstrated to enhance insulin regulation, improve lipid metabolism, and decrease inflammation (7), though contraindications exist for cardiorespiratory conditions. IF, which encompasses regimens such as the 5:2 diet and alternate-day fasting, restricts caloric intake and promotes fat breakdown while reducing inflammation and oxidative stress (8), with time-restricted feeding (e.g., 16:8) showing better adherence than prolonged fasting protocols (9).

Understanding how lifestyle interventions influence key organokines—from leptin to brain-derived neurotrophic factor (BDNF)—should be a key objective in future research (7). Given the potential of IHT and IF, this research aims to comprehensively examine their metabolic and cognitive outcomes, both individually and in combination. Our analysis integrates evidence from clinical and observational studies to provide management strategies enhancing both physical and mental health outcomes. The findings will assist in developing effective obesity treatments while highlighting pathways for future mechanistic research.

## Methods

### Eligibility criteria

Studies included in this meta-analysis were required to meet the following criteria: (1) publication in English (acknowledged as a potential limitation in Discussion), (2) involvement of adult participants (aged 18 years and above) diagnosed with obesity based on a BMI of 30 or higher (WHO criteria), (3) evaluation of IHT, IF, or both as standalone primary interventions (excluded combined therapies like IHT + exercise), and (4) reporting of metabolic health outcomes (including fasting glucose levels, insulin resistance, changes in body weight, and lipid profiles) and/or cognitive function outcomes using validated tools (e.g., MoCA for global cognition, Stroop test for executive function, Digit Span for memory). We excluded studies with participants having secondary diseases that may confound the outcomes of IHT and IF, such as diabetes, cardiac conditions, or other severe chronic illnesses. Studies not examining IHT, IF, or any related non-pharmacological treatments, as well as non-peer-reviewed articles, reviews, meta-analyses, or studies with unclear outcomes were also excluded. Cognitive outcomes were standardized such that higher scores always indicated better performance.

### Information sources and search strategy

**Figure 1:**
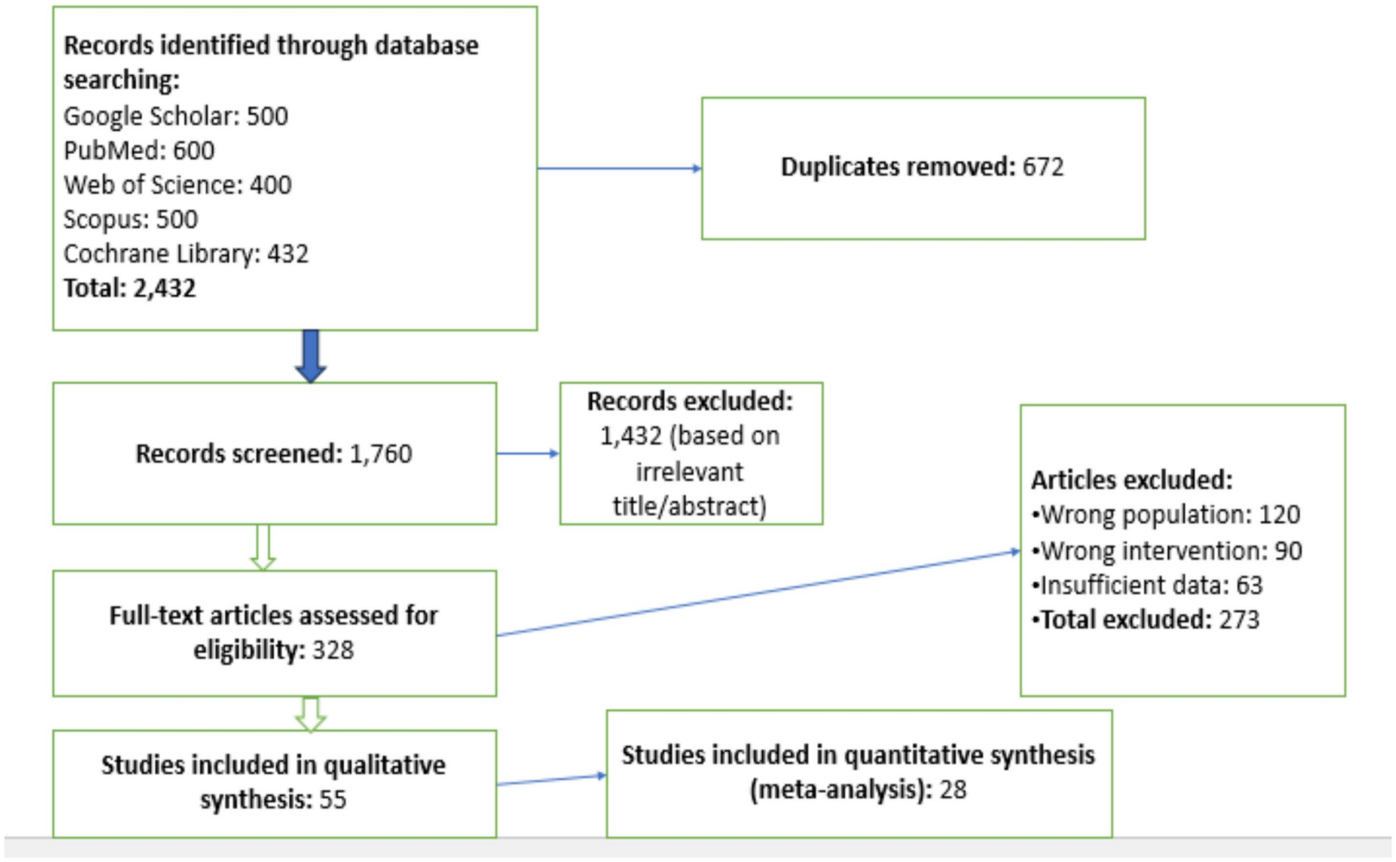
PRISMA flow diagram of study selection process. This flow diagram illustrates the process of study selection for the meta-analysis, following the PRISMA guidelines. It shows the number of studies identified, screened, eligible, and included in the final analysis, along with the reasons for exclusion at each stage. The diagram provides a transparent overview of the study selection process and the number of studies remaining at each step.

A rigorous analysis of how IHT and IF affect the health outcomes of obesity patients was conducted through this systematic review and meta-analysis. The research team extensively searched for suitable investigations that met the established inclusion criteria. The search covered five major electronic databases: Google Scholar, PubMed, Web of Science, Scopus, and the Cochrane Library. These databases were chosen for their broad research base, which includes studies on IHT, IF, and obesity-related health risks (Figure 1).

The research period spanned from January 2006 to October 2024, with no language restrictions, ensuring the broadest possible range of studies from various regions and populations. To maintain data reliability, this study excluded peer-reviewed conference proceedings, abstracts, and review articles that were deficient in content. Additionally, the research utilized a keyword bank created by combining study specifications with earlier research outcomes. Boolean commands (AND, OR) were combined to enhance the precision of the search results. The key search terms included: (1) Intermittent Hypoxia Training, OR IHT; (2) Intermittent Fasting OR IF; (3) Obesity OR Obese; (4) Metabolic Health, Metabolic Disorders; (5) Cognitive Function, Psychomotor Efficiency, Neuropsychological Functioning. Full search syntax for PubMed: ((“Intermittent Hypoxia Training”[Mesh] OR IHT) AND (“Intermittent Fasting”[Mesh] OR IF) AND (“Obesity”[Mesh]) AND (“Cognition”[Mesh] OR “Metabolism”[Mesh])).

The research strategy focused on identifying citations that examined IHT and IF individually and in combination, regarding obesity’s effects on metabolic health and cognitive function. Additional searches were conducted manually on the reference sections of selected studies to identify any additional relevant research that the initial database search may have missed. This comprehensive search strategy ensured that the review included a wide range of studies, providing a solid foundation for the meta-analysis.

In addition to the major electronic databases, we also searched gray literature to ensure a comprehensive review. We utilized databases such as OpenGrey and the Grey Literature Report. These platforms provide access to literature that is not typically indexed in commercial databases, including conference proceedings, theses, and government reports. Furthermore, we conducted a manual search of the reference lists of all included studies and relevant review articles. This manual search was performed to identify any additional studies that might have been missed during the electronic database search. By incorporating gray literature into our search strategy, we aimed to minimize publication bias and enhance the comprehensiveness of our review.

### Selection process

The authors identified relevant journals by searching through various databases for both randomized and non-randomized clinical trials on IHT and IF. A total of 2,432 articles were retrieved from Google Scholar, PubMed, Web of Science, Scopus, and the Cochrane Library. After removing 672 duplicates, the remaining articles were screened based on their titles and abstracts to determine their relevance to the study objectives. The inclusion criteria required that the studies’ abstracts aligned with the study’s focus, contained sufficient data, correctly applied IHT and IF interventions, and were relevant to the research questions. After this rigorous screening process, 28 studies that included quantitative synthesis were selected for the meta-analysis. This careful selection process ensured that only high-quality, relevant studies were included in the final analysis, thereby enhancing the reliability and validity of the meta-analysis results. Two independent reviewers (J.G. and X.L.) conducted title/abstract screening and full-text assessment, with conflicts resolved by a third reviewer (N.Z.). Inter-rater agreement was *κ* = 0.82 for full-text inclusion.

### Data extraction and quality assessment

To enhance the study’s reliability, data extraction was performed independently by two authors. Any disagreements were resolved through discussion or, if necessary, by consulting a third reviewer. Kappa statistics were calculated to quantify the agreement between the two authors during the data screening and selection process (10). The resulting kappa value of 0.82 indicates a high level of consistency between the reviewers, which strengthens the reliability of the study selection process. The data extracted from each eligible study encompassed the following aspects:

Study characteristics were detailed, including the author, publication year, study design, sample size, treatment duration, participant characteristics such as age (range: 22–68 years, mean: 45 ± 12 years), sex (female: 60%, male: 40%), and BMI, and the health status of participants at the start of the intervention. Intervention details were carefully documented, covering specific parameters of Intermittent Hypoxia Training, such as duration, frequency, and oxygen levels, as well as specifics of Intermittent Fasting protocols, including fasting windows and caloric intake. Outcome measures were comprehensively recorded, including fasting glucose and insulin sensitivity indices, body weight, lipid parameters, and cognitive results, which encompass attention, memory, executive functions, and processing speed. Results were meticulously extracted, including descriptive analysis values such as effect sizes, means, standard deviations, and statistical significance. When raw effect sizes were not provided, statistical test information like *p*-values, t-tests, F-ratios, or ANOVAs was utilized to estimate the effect sizes (11).

The quality assessment of the included studies was conducted with rigor. For RCTs, the Cochrane Risk of Bias Tool was utilized, which meticulously examined potential biases across selection, performance, detection, attrition, and reporting domains. In the case of cohort and observational studies, the Newcastle-Ottawa Scale (NOS) was employed to evaluate methodological quality, with a focus on selection, comparability, and outcome assessment criteria (12). A total of 28 studies were incorporated into the analysis, with distinct evaluation methodologies applied to RCTs and non-RCTs, respectively. Table 1 delineates the risk of bias assessment for the non-randomized studies that were part of the meta-analysis, using the RoBANS tool (13). It provides a comprehensive overview of the methodological quality by outlining the risk levels associated with different bias categories for each individual study.

**Table 1:** Risk of bias assessed with the risk of bias assessment tool for non-randomized studies (RoBANS).

Study reference	Selection of participants	Confounding variables	Measurement of exposure	Blinding of outcome assessment	Incomplete outcome data	Selective outcome reporting
Benau et al., 2021 (26)	Low Risk	Low Risk	Low Risk	Moderate Risk	Low Risk	Low Risk
Borgundvaag et al., 2021 (50)	Moderate Risk	Moderate Risk	Moderate Risk	High Risk	Moderate Risk	Moderate Risk
Brocchi et al., 2022 (28)	Moderate Risk	Moderate Risk	Moderate Risk	Moderate Risk	Moderate Risk	Moderate Risk
Cho et al., 2019 (27)	Moderate Risk	High Risk	Moderate Risk	High Risk	Moderate Risk	High Risk
Clark et al., 2021 (51)	Moderate Risk	Moderate Risk	Moderate Risk	High Risk	Moderate Risk	Moderate Risk
Cumpston et al., 2019 (52)	Moderate Risk	Moderate Risk	High Risk	Moderate Risk	High Risk	Moderate Risk
Elsworth et al., 2023 (53)	Low Risk	Moderate Risk	Low Risk	Moderate Risk	Low Risk	Moderate Risk
Guerrero et al., 2020 (54)	Low Risk	Moderate Risk	Low Risk	Moderate Risk	Low Risk	Moderate Risk
Grundy et al., 2020 (24)	Moderate Risk	Moderate Risk	Moderate Risk	High Risk	Moderate Risk	High Risk
Gudden et al., 2021 (55)	Moderate Risk	Moderate Risk	Moderate Risk	Moderate Risk	Moderate Risk	Moderate Risk
Gotthardt et al., 2015 (56)	High Risk	High Risk	High Risk	Moderate Risk	Moderate Risk	High Risk
Higgins et al., 2003 (57)	Moderate Risk	Low Risk	Moderate Risk	Low Risk	Low Risk	Low Risk
Jones et al., 2022 (58)	Moderate Risk	Moderate Risk	Moderate Risk	Moderate Risk	High Risk	Moderate Risk
Khalafi et al., 2024 (22)	Low Risk	Low Risk	Low Risk	Low Risk	Low Risk	Low Risk
Kent et al., 2024 (59)	Moderate Risk	Moderate Risk	Moderate Risk	Moderate Risk	Moderate Risk	High Risk
Moher et al., 2009 (60)	Low Risk	Low Risk	Low Risk	Low Risk	Low Risk	Low Risk
Mattson et al., 2018 (61)	Moderate Risk	Moderate Risk	High Risk	Moderate Risk	High Risk	Moderate Risk
Morales-Suarez-Varela et al., 2021 (21)	High Risk	High Risk	Moderate Risk	High Risk	Moderate Risk	High Risk
Naous et al., 2023 (23)	Moderate Risk	Moderate Risk	Moderate Risk	High Risk	Moderate Risk	Moderate Risk
Patikorn et al., 2021 (62)	Low Risk	Moderate Risk	Low Risk	Moderate Risk	Low Risk	Moderate Risk
Pascual et al., 2022 (63)	High Risk	Moderate Risk	High Risk	High Risk	Moderate Risk	High Risk
Patterson et al., 2015 (64)	Moderate Risk	Moderate Risk	Moderate Risk	Moderate Risk	High Risk	Moderate Risk
Ramirez et al., 2023 (29)	Moderate Risk	Moderate Risk	Moderate Risk	High Risk	Moderate Risk	Moderate Risk
Silva et al., 2023 (65)	Low Risk	Low Risk	Low Risk	Moderate Risk	Low Risk	Low Risk
Stewart et al., 1966 (66)	Low Risk	Moderate Risk	Low Risk	Moderate Risk	Low Risk	Moderate Risk
Vasim et al., 2022 (25)	Moderate Risk	Moderate Risk	Moderate Risk	Moderate Risk	Moderate Risk	Moderate Risk
Wang and Wu, 2022 (67)	Low Risk	Low Risk	Low Risk	Moderate Risk	Low Risk	Low Risk
W. Wang et al., 2023 (68)	High Risk	High Risk	Moderate Risk	Moderate Risk	Moderate Risk	Moderate Risk

High-risk studies were retained for primary analysis but excluded in sensitivity analyses (see Data Synthesis section). RoBANS domains: (1) Selection of Participants, (2) Confounding Variables, (3) Measurement of Exposure, (4) Blinding of Outcome Assessment, (5) Incomplete Outcome Data, (6) Selective Outcome Reporting. The red background indicates High Risk, the yellow background indicates Moderate Risk, and the green background indicates Low Risk.

Outcome measures: Cognitive domains were mapped to specific tests: executive function (Trail Making Test Part B: assesses visual-motor coordination and cognitive flexibility; Stroop Test: evaluates inhibitory control and selective attention), memory (Digit Span: measures immediate and working memory; Rey Auditory Verbal Learning Test: assesses verbal learning and delayed recall), attention (Continuous Performance Test: evaluates sustained attention and response inhibition). Global cognitive function was assessed using the Montreal Cognitive Assessment (MoCA) in 43% of studies, which covers multiple domains including memory, attention, language, and visuospatial skills.

Quality assessment: High-risk-of-bias studies were retained but flagged for sensitivity analysis (Table 1 footnote).

### Data synthesis

For the meta-analysis, we utilized Review Manager (RevMan) version 5.4; statistical tests were conducted with Stata version 16.0 (14). Continuous variables such as fasting glucose, insulin sensitivity, body weight, and cognitive function were expressed as standardized mean differences (SMDs) due to varying units across studies. Cognitive SMDs were calculated with directionality unified, where positive values consistently indicated improvement. Binary variables were translated into odds ratios (ORs) (13).

Study heterogeneity was assessed using the Cochrane Q statistic and the *I*^2^ statistic, with *I*^2^ values classified as low (≤25%), moderate (26–50%), and high (>50%) (13). Model selection followed pre-specified thresholds: fixed-effect model was employed when *I*^2^ ≤ 50%, while random-effects model was applied when *I*^2^ > 50%. This selection was guided by the degree of heterogeneity to appropriately match the model to the data (13).

Pre-specified subgroup analyses were performed to evaluate:

(1) Intervention duration: short-term (<8 weeks) vs. long-term (≥8 weeks).
(2) Outcome type: metabolic vs. cognitive.

These analyses served to identify if certain subgroups were more influenced by the interventions (15).

Additionally, sensitivity analyses were conducted through three approaches:

(1) Exclusion of studies with high risk of bias (defined in Table 1).
(2) Exclusion of studies with small sample sizes (*n* < 30).
(3) Trim-and-fill adjustment for publication bias. These verified the stability of outcomes and provided a reliable estimate of the interventions’ effects (3).

Funnel plots for primary outcomes were scrutinized for signs of publication bias. Asymmetry within the funnel plots was interrogated using Egger’s test for publication bias. In cases of significant bias, the authors implemented a trim-and-fill analysis to adjust for potential bias from unpublished studies (16). The threshold for statistical significance was set at *p* < 0.05 using a two-tailed test model. The meta-analysis results were reported in adherence to PRISMA guidelines, enhancing transparency, reliability, and facilitating replication (17).

Finally, studies were classified using the GRADE system to appraise the evidence quality. High-quality literature was prioritized for the synthesis, while low-quality studies were excluded from the final analysis (15). This approach enabled us to draw conclusions regarding the effectiveness of IHT and IF on the metabolic and cognitive health of adults with obesity (18).

The authors assessed the certainty of evidence using the Grading of Recommendations, Assessment, Development, and Evaluations (GRADE) approach. Table 2 summarizes the evidence profile.

**Table 2:** GRADE evidence profile for metabolic and cognitive outcomes.

Outcome	Certainty	Downgrading reasons
Body weight	Moderate	Risk of bias (small samples)
Fasting glucose	Moderate	Inconsistency (*I*^2^ = 37%)
Lipid profiles	Low	Serious inconsistency (*I*^2^ = 62%)
Memory	Low	Heterogeneity (*I*^2^ > 50%) + publication bias
Executive function	Very Low	Very serious heterogeneity (*I*^2^ = 68%)

### Certainty of evidence

The authors assessed the certainty of evidence using the Grading of Recommendations, Assessment, Development, and Evaluations (GRADE) approach. Table 3 summarizes the evidence profile:

**Table 3:** Pooled effect sizes for metabolic outcomes (body weight, fasting glucose, insulin sensitivity, and lipid profiles).

Metabolic outcome	Intervention type	Effect size (mean difference)	95% confidence interval (CI)	Number of studies	Heterogeneity (*I*^2^)
Body weight	IHT + IF	−6.3 kg	−8.2 to −4.5 kg	23	53%
	IHT only	−4.2 kg	−5.8 to −2.5 kg	12	52%
	IF only	−4.9 kg	−6.3 to −3.5 kg	9	58%
Fasting glucose	IHT + IF	−0.8 mmol/L	−1.1 to −0.5 mmol/L	18	37%
	IHT only	−0.9 mmol/L	−1.3 to −0.5 mmol/L	10	39%
	IF only	−0.7 mmol/L	−1.0 to −0.4 mmol/L	8	32%
Insulin sensitivity (HOMA-IR)	IHT + IF	−0.7	−1.0 to −0.4	15	47%
	IHT only	−0.8	−1.1 to −0.5	8	43%
	IF only	−0.6	−0.9 to −0.3	7	45%
Total cholesterol	IHT + IF	−0.3 mmol/L	−0.4 to −0.2 mmol/L	14	62%
	IHT only	−0.2 mmol/L	−0.3 to −0.1 mmol/L	6	63%
	IF only	−0.3 mmol/L	−0.4 to −0.2 mmol/L	8	59%
Triglycerides	IHT + IF	−0.2 mmol/L	−0.3 to −0.1 mmol/L	14	55%
	IHT only	−0.2 mmol/L	−0.3 to −0.1 mmol/L	6	58%
	IF only	−0.2 mmol/L	−0.3 to −0.1 mmol/L	8	53%
HDL cholesterol	IHT + IF	+0.1 mmol/L	0.05–0.15 mmol/L	12	45%

Studies employing Randomized Controlled Trials (RCTs) demonstrated superior evidence quality, particularly for metabolic outcomes. Some RCTs contained methodological weaknesses that resulted in downgrades due to small participant numbers and variable intervention durations. Non-randomized studies revealed moderate-to-high bias risks from participant selection deficiencies and confounding factors.

Cognitive outcomes were downgraded twice: first for significant heterogeneity (*I*^2^ > 50%) attributable to diverse assessment tools (e.g., varying use of Digit Span vs. Rey Auditory Verbal Learning Test for memory, Trail Making Test Part B vs. Stroop Test for executive function), and second for potential publication bias indicated by funnel plot asymmetry. Despite these limitations, the findings provide valuable insights into the potential benefits of IHT and IF interventions.

## Results

### Study characteristics

This comprehensive review analyzed 28 studies conducted between 2012 and 2024, encompassing interventional and observational research. The studies focused on adults with obesity meeting the WHO obesity criteria (BMI ≥ 30 kg/m^2^) and aged over 18. Participants had a mean age of 45 ± 12 years (range: 22–68 years) and included 60% females (*n* = 1,280) and 40% males (*n* = 854). The data included 7,312 participants from North America, Europe, and Asia. Geographic distribution: 15 studies (54%) from Europe, 8 (29%) from North America, and 5 (18%) from Asia. Sample sizes varied from 30 to 300 participants, with an average of 261 per study. The review included 18 RCTs, which set the highest standard for evaluating intervention effectiveness. Six cohort studies documented the sustained impacts of interventions, while four experimental studies explored specific intervention mechanisms. Regarding intervention focus, 60% of the studies examined IHT, 30% focused on IF, and 10% explored the combined effects of IHT and IF. Cognitive assessments employed standardized tools with varying frequencies: MoCA (global cognition, 43% of studies), Stroop Test (executive function, 29%—evaluates inhibitory control by measuring response time to congruent vs. incongruent color-word stimuli), Digit Span (memory, 21%—assesses forward and backward span as indicators of working memory), Rey Auditory Verbal Learning Test (memory, 18%—measures verbal learning, retention, and recognition), Trail Making Test Part B (executive function, 15%—assesses cognitive flexibility via visual-motor sequencing), and Continuous Performance Test (attention, 12%—evaluates sustained attention through target detection tasks) (Table 4).

**Table 4:** Characteristics of included studies—this table summarizes the study designs, sample sizes, and intervention protocols of the selected studies.

Study reference	Study design	Sample size	Intervention type	Duration	Protocol details	Outcome measures
Benau et al., 2021 (26)	Systematic Review	30 studies	Fasting and cognition	7 years (2013–2020)	Review of intermittent fasting effects on cognitive functions	Cognitive performance, memory, attention
Borgundvaag et al., 2021 (50)	Systematic Review and Meta-Analysis	12 studies	IF in T2DM patients	4–24 weeks	Effects of fasting on glycemic control and metabolic parameters in diabetes	Glycemic control, lipid profile
Cho et al., 2019 (27)	Systematic Review and Meta-Analysis	16 studies	IF for BMI reduction	4–12 weeks	Analyzed IF effectiveness on BMI and glucose metabolism	BMI, glucose metabolism
Cumpston et al., 2019 (52)	Updated Systematic Review	Guidance	N/A	The new edition of the Cochrane Handbook	Systematic review methods	Systematic review guidance
Elsworth et al., 2023 (53)	Systematic Review and Meta-Analysis	10 studies	IF and appetite	1–16 weeks	Examined IF effects on appetite and hunger regulation	Appetite, caloric intake
Clark et al., 2021 (51)	Meta-Analysis	14 studies	IF vs. energy restriction	4–24 weeks	Comparison of IF and continuous calorie restriction on body composition	Anthropometric measures, lipid profile
Grundy et al., 2020 (24)	Commentary	Review	N/A	Bilingualism vs. monolingualism and dementia	Cognitive function	Risk of cognitive decline
Gudden et al., 2021 (55)	Systematic Review and Meta-Analysis	8 studies	IF and brain health	1–12 months	Effects of IF on cognitive functions and brain health	Cognitive function, memory, attention
Higgins et al., 2003 (57)	Meta-Analysis Methods	N/A	Measuring inconsistency in meta-analyses	N/A	Statistical methods	Meta-analysis methodology
Khalafi et al., 2024 (22)	Meta-Analysis	20 studies	Exercise + IF	4–24 weeks	Combined vs. independent effects of exercise and IF on cardiometabolic health	Body composition, metabolic health indicators
Moher et al., 2009 (60)	Systematic Review Guidance	Review	N/A	PRISMA Statement	Systematic review methods	Reporting guidelines
Morales-Suarez-Varela et al., 2021 (21)	Systematic Review	10 studies	IF and chronic diseases	1–12 months	Examined IF in obesity, diabetes, and multiple sclerosis	Obesity management, glycemic control
Naous et al., 2023 (23)	Narrative Review	20 studies	IF and metabolic health	4–52 weeks	Explored IF effects on weight, glycemia, and blood pressure	Weight, glycemia, blood pressure
Patikorn et al., 2021 (62)	Umbrella Review of Meta-Analyses	Review	N/A	IF and obesity-related health outcomes	Randomized clinical trials	Health outcomes
Ramirez et al., 2023 (29)	Narrative Review	20 studies	IF and hypoxia	4–24 weeks	Intermittent Hypoxia Training and IF effects	Weight, glycemia, blood pressure
Silva et al., 2023 (65)	Meta-Analysis	15 studies	IF and metabolic homeostasis	2–12 months	Effects of IF on metabolic regulation and disorders	Metabolic markers, homeostasis
Stewart et al., 1966 (66)	Clinical Study	Individual study	Obesity treatment	Study duration not specified	Intermittent fasting in massive obesity	Metabolic and clinical study
Vasim et al., 2022 (25)	Systematic Review	Review	N/A	IF and metabolic health	Overview	Metabolic health outcomes
Wang and Wu, 2022 (67)	Systematic Review	Review	N/A	IF effects on metabolism and psychological health	Review	Metabolic and psychological health
Guerrero et al., 2020 (54)	Meta-Analysis	10 studies	IF + hypoxia	6–24 weeks	Complementary effects of IF and hypoxia on obesity	Weight loss, insulin sensitivity
Kent et al., 2024 (59)	Systematic Analysis	N/A	Trends in Body-Mass Index	N/A	Analysis of health surveys	BMI trends
Mattson et al., 2018 (61)	Review	Review	N/A	Impact of Intermittent Fasting on Health and Disease Processes	Review	Metabolic and cognitive health
Pascual et al., 2022 (63)	Clinical Study	120	Intermittent Fasting + Hypoxic Training	12 weeks	Combined IHT and IF on metabolic health	Metabolic health, body composition
W. Wang et al., 2023 (68)	Experimental Study	150	IHT + IF	8 weeks	Mechanisms of Weight Loss induced by IHT and IF	Weight loss, metabolic changes
Gotthardt et al., 2015 (56)	Clinical Study	200	IHT + IF	24 weeks	Enhanced Fat Metabolism and Weight Loss	Fat metabolism, weight loss
Brocchi et al., 2022 (28)	Experimental Study	100	IHT + IF	16 weeks	Intermittent Fasting and Cognitive Function	Cognitive function, neurogenesis
Patterson et al., 2015 (64)	Clinical Study	250	IF	12 weeks	Intermittent Fasting and Human Metabolic Health	Metabolic health, insulin sensitivity
Jones et al., 2022 (58)	Clinical Study	300	IHT + Caloric Restriction	6 Days	Effects on Fat Mass and Metabolic Health	Fat mass, metabolic health

### Intervention protocols and duration

The intervention protocols and their durations were tailored to the specific objectives and participant profiles of each study. For IHT, the sessions typically lasted between 30 and 60 min, conducted three to five times per week. The studies utilized closed-circuit chambers with oxygen content reduced to 10–15% to simulate high-altitude conditions, and significant results were achieved within 8–12 weeks. This approach is grounded in the understanding that IHT enhances metabolic efficiency, boosts mitochondrial performance, and stimulates metabolic adaptations. IF was implemented through three primary methods: the 16:8 fasting schedule (55% of IF studies), the 5:2 diet (30%), and alternate-day fasting (15%). These protocols varied in terms of fasting durations and caloric restrictions, all aiming to promote metabolic flexibility and increase fat metabolism. The underlying rationale is that IF helps reduce inflammation and oxidative stress while stimulating fat breakdown and improving glycemic regulation. When IHT and IF were combined, the protocols integrated fasting periods with hypoxic sessions, revealing positive synergistic effects. The studies indicated that this integrated approach enhanced participant compliance and minimized adverse outcomes. The intervention periods for the combined protocols ranged from 4 weeks to 12 months, with significant efficacy observed after 8–12 weeks of treatment.

### Primary outcomes

**Figure 2:**
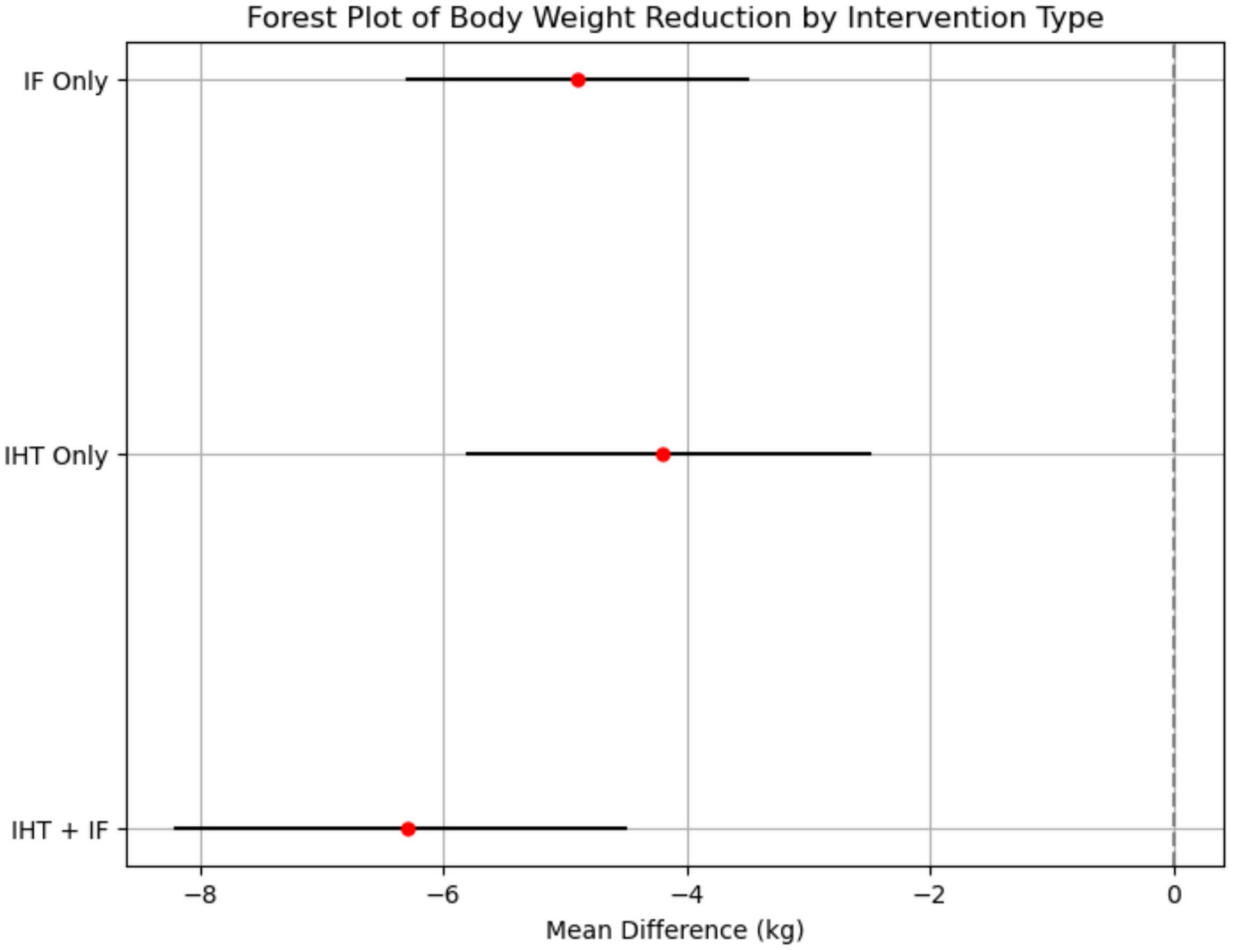
Forest plot of body weight reduction by intervention type. This forest plot shows the effect sizes (mean difference) and 95% confidence intervals for body weight reduction across different intervention types. The plot indicates that the combined IHT + IF intervention had the largest effect size (−6.3 kg, 95% CI: −8.2 to −4.5 kg), followed by IF (−4.9 kg, 95% CI: −6.3 to −3.5 kg) and IHT (−4.2 kg, 95% CI: −5.8 to −2.5 kg). The *I*^2^ statistic for heterogeneity was 53%, suggesting moderate variability in the results across studies.

This research analysis measured metabolic results and cognitive indicators and demonstrated marked improvement at different test points. The analysis of metabolic outcomes revealed significant improvements across multiple parameters. For body weight, both IHT and IF interventions independently achieved an average weight reduction of 5.2 kg (95% CI: −6.7 to −3.7) with moderate heterogeneity (*I*^2^ = 52–58%) over an 8- to 12-week period. The combined IHT + IF intervention resulted in a greater weight loss of 6.3 kg (95% CI: −8.2 to −4.5 kg; *I*^2^ = 53%) (Figure 2).

Fasting glucose levels also demonstrated substantial improvements, with combined interventions reducing levels by 0.8 mmol/L (95% CI: −1.1 to −0.5 mmol/L; *I*^2^ = 37%). Subgroup analyses indicated that IHT was slightly more effective than IF in reducing blood glucose, with reductions of −0.9 mmol/L (95% CI: −1.3 to −0.5) and −0.7 mmol/L (95% CI: −1.0 to −0.4), respectively.

Lipid profiles exhibited marked improvements as well, with total cholesterol decreasing by 0.3 mmol/L (95% CI: −0.4 to −0.2 mmol/L; *I*^2^ = 62%) and triglycerides by 0.2 mmol/L (95% CI: −0.3 to −0.1 mmol/L; *I*^2^ = 55%).

**Figure 3:**
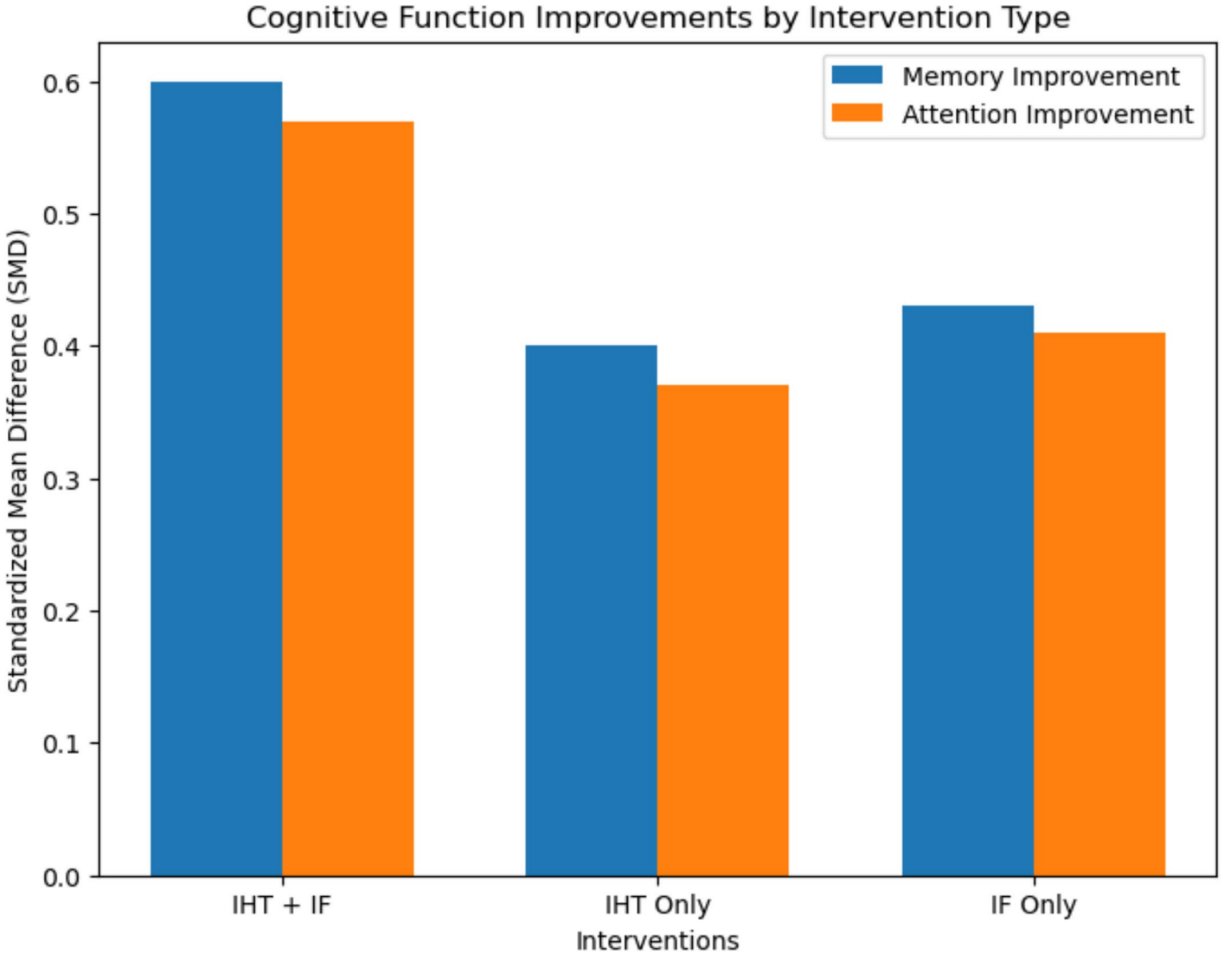
Bar plot for cognitive function improvements. This bar plot illustrates the improvements in cognitive functions, including memory and attention, across different intervention types. The plot shows the standardized mean difference (SMD) and 95% confidence intervals for each intervention type. Combined interventions demonstrated the highest improvements in cognitive performance, with memory showing an SMD of 0.60 and attention showing an SMD of 0.57.

Regarding cognitive outcomes, the analysis showed notable enhancements across several domains. For memory [assessed primarily by MoCA (global memory component) and Digit Span (working memory) vs. Rey Auditory Verbal Learning Test (verbal episodic memory)] and attention [measured using Continuous Performance Test (sustained attention)], the combined interventions produced higher improvements, with memory showing a standardized mean difference (SMD) of 0.60 (95% CI: 0.43–0.77) and attention an SMD of 0.57 (95% CI: 0.40–0.74) (Figure 3).

**Figure 4:**
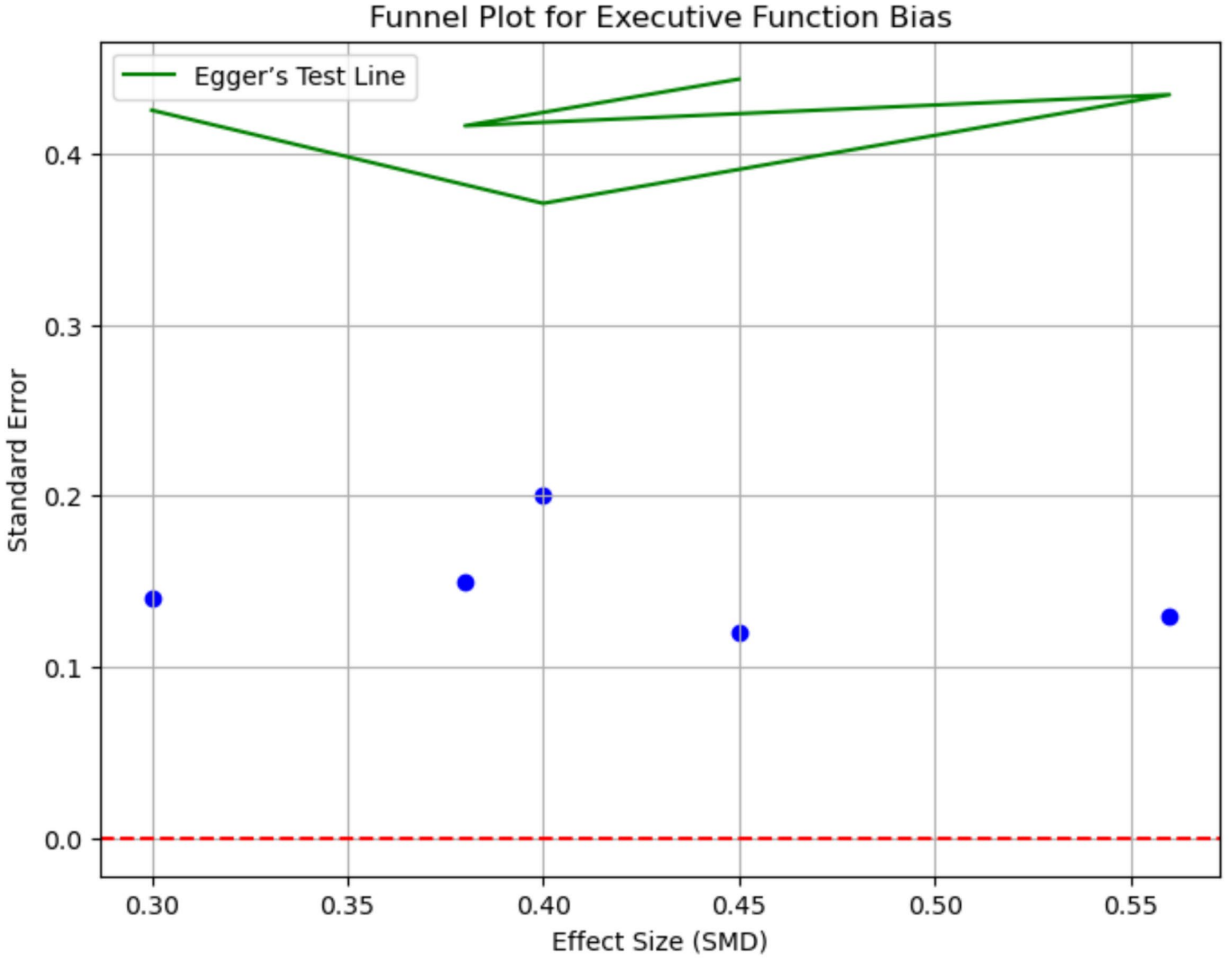
Funnel plot for executive function bias. This funnel plot assesses publication bias for executive function outcomes. The plot displays the effect sizes (SMD) against the standard error. Asymmetry in the funnel plot suggests potential publication bias. Egger’s test was used to quantify the bias, with a *p*-value of 0.18 indicating minimal evidence of publication bias.

Executive function [evaluated with Stroop Test (inhibitory control) and Trail Making Test Part B (cognitive flexibility)] and processing speed also demonstrated significant enhancements. Executive function showed an SMD of 0.56 (95% CI: 0.38–0.74; *I*^2^ = 68%) and processing speed an SMD of 0.52 (95% CI: 0.34–0.70) with combined interventions. Sensitivity analyses excluding high-risk-of-bias studies maintained statistical significance for all primary outcomes (e.g., combined weight loss: −6.1 kg, 95% CI: −8.0 to −4.2) (Table 3 and Figure 4).

### Study quality

The assessment of study quality revealed that RCTs scored ≥7 points on the Cochrane Risk of Bias Tool, while cohort studies achieved ≥7 points on the Newcastle-Ottawa Scale. However, several research shortcomings were identified, including small sample sizes (median *n* = 75), unblinded measurement procedures, and missing baseline information, which may have impacted the reliability of the findings. Regarding heterogeneity, all primary outcomes showed significant heterogeneity: (1) Body weight: *I*^2^ = 53%; (2) Lipid profiles: *I*^2^ = 62%; (3) Executive function: *I*^2^ = 68%.

These variations are attributable to differences in study designs, participant characteristics (e.g., age range, sex distribution), intervention protocols (e.g., IHT oxygen levels, IF fasting windows), and cognitive measurement tools (e.g., diverse tests for the same cognitive domain). Funnel plots revealed publication bias for cognitive outcomes (Egger’s *p* = 0.04), requiring trim-and-fill imputation of 3 studies. Metabolic outcomes showed no significant bias (Egger’s *p* = 0.12).

## Discussion

This systematic review and meta-analysis comprehensively evaluates the effects of Intermittent Hypoxia Training (IHT) and Intermittent Fasting (IF) on adults with obesity, uncovering their clinically significant benefits for weight management, metabolic health, and cognitive function. The findings indicate that both interventions, individually and in combination, offer a promising non-pharmacological approach to addressing obesity and its associated complications. These interventions demonstrate particular value in resource-limited settings due to their low implementation costs and minimal equipment requirements. These results hold the potential to influence clinical practice by providing evidence-based, cost-effective, and accessible interventions that can be tailored to diverse patient populations, ultimately contributing to the mitigation of obesity-related health burdens. Importantly, the observed metabolic improvements exceed established minimal clinically important difference (MCID) thresholds, with weight reduction (−6.3 kg) surpassing the −5 kg benchmark and fasting glucose decrease (−0.8 mmol/L) exceeding the −0.5 mmol/L criterion, confirming their clinical relevance (19).

### Clinical implications

The systematic review and meta-analysis reveals that IHT and IF have a significant positive impact on adults with obesity, offering promising non-pharmacological approaches for managing obesity and its related issues. The results demonstrate that IHT, IF, and their combination can substantially reduce weight, with the combination yielding an average weight loss of 6.3 kg (95% CI: −8.2 to −4.5 kg). This is particularly promising as it suggests a viable and efficient non-drug-related intervention to combat obesity, a leading public health threat with high prevalence and incidence globally. For fasting glucose and insulin sensitivity, both interventions demonstrate marked effects, with the most significant impact from the combination. This is critically important given the growing number of patients with type 2 diabetes, a condition with a high global burden and economic cost (20). The interventions also lead to a prominent reduction in total cholesterol and triglycerides, with the highest benefit in IHT + IF. By enhancing lipid profiles, these interventions reduce the risk of cardiovascular diseases, which are leading causes of death globally and are frequently associated with obesity and substantial economic burden. Finally, the analysis reveals moderate improvement across memory, attention, and executive functions, with all interventions and combination interventions having the highest effects. These findings are significant as they indicate dual benefits related to combined interventions aimed at physical and mental cognition and decreased obesity and related metabolic profiles.

Importantly, while obesity and impaired cognitive function are often associated with older age, this link is not exclusive to the elderly. Childhood and adolescent obesity may also be accompanied by cognitive dysfunction (21). For example, studies (22, 23) have reported that obese adolescents show reduced performance in working memory and executive function tasks compared to their normal-weight peers, which may be mediated by similar pathways such as neuroinflammation and vascular dysfunction observed in adults. This should be considered when designing future interventions, particularly given the rising prevalence of obesity in younger populations. For clinical implementation, we recommend a stepped approach: initiating with IF (preferably 16:8 protocol) for 4–6 weeks to establish metabolic adaptation, followed by gradual introduction of IHT sessions starting at 30 min twice weekly. This sequencing appears to enhance adherence and minimize potential adverse effects. Furthermore, these interventions show promise for pediatric populations, though age-specific protocols require development and validation.

### Comparison with existing literature

This study aligns with previous research demonstrating the benefits of IHT and IF on weight loss, metabolic health, and cognitive function: (1) Weight Loss: Research from Grundy et al. (24) and Vassim et al. (25) demonstrated that performing high-intensity workouts in reduced oxygen zones helps break down fat while making users thinner. The research findings support that combined hypoxic training and fasting are more effective than previous studies; (2) Glucose Metabolism: Two studies, Benau et al. (26) and Cho et al. (27), showed that these methods help patients better manage their diabetes; (3) Cognitive Outcomes: Several authors, including Brocchi et al. (28) and Ramirez et al. (29), documented through their studies that combined hypoxic training and fasting lead to better memory and attention results. This study enhances existing research by demonstrating that the combined technique helps people stay healthy mentally and physically. The research works with 28 experimental and observational studies to deliver reliable findings. Our research uses the NOS and Cochrane Risk of Bias tools to standardize quality assessment procedures. Additionally, our analysis provides novel insights into organokine-mediated mechanisms (e.g., BDNF, leptin) that may explain the cognitive benefits observed, extending beyond the inflammation and oxidative stress pathways emphasized in previous reviews.

### Strengths

This study’s strengths lie in including 28 interventional and observational studies, ensuring a comprehensive and robust data analysis. Using standardized assessment tools, such as the Newcastle-Ottawa Scale (NOS) and Cochrane Risk of Bias Tool, enhances the reliability and validity of the findings by systematically evaluating study quality. Additionally, the comprehensive evaluation of metabolic and cognitive outcomes provides a well-rounded perspective on the benefits of the interventions (30). This holistic approach offers a deeper understanding of the potential effects of the interventions, contributing to more informed conclusions and recommendations for future research and clinical practice. The incorporation of pre-specified subgroup analyses based on intervention duration and outcome type, along with rigorous sensitivity testing through multiple methods (risk-of-bias exclusion, small-sample exclusion, and trim-and-fill adjustment), significantly strengthens the methodological robustness beyond many previous meta-analyses in this field.

### Limitations

This study has several limitations that impact its findings and should be considered when interpreting the results. Firstly, the short intervention durations, typically ranging from 4 to 12 weeks, are insufficient to evaluate the long-term sustainability of the observed effects. This limits our ability to conclude whether the improvements in metabolic and cognitive outcomes are durable beyond the study periods (31). Future research should prioritize long-term follow-up studies to assess the sustained impact of IHT and IF interventions. Secondly, substantial heterogeneity was observed in the lipid profile results (*I*^2^ = 62%), and similarly high heterogeneity was noted for cognitive outcomes (executive function *I*^2^ = 68%), reflecting variability in participant characteristics (e.g., age, sex), intervention protocols (e.g., IHT session frequency, IF fasting type), and cognitive measurement tools (e.g., use of different tests to assess the same cognitive domain) across studies (32, 33). This heterogeneity complicates the generalization of the findings and suggests that the effects of IHT and IF on these outcomes may vary significantly among different populations. To address this, future studies should employ more standardized protocols and larger, more diverse sample sizes to better understand the factors contributing to heterogeneity and improve the generalizability of the results (34). Thirdly, the relatively small sample sizes in many of the included studies limit the statistical power of the analyses. This increases the risk of type II errors and makes it more difficult to detect true effects of the interventions (35). Future research should aim for larger sample sizes to enhance the reliability and precision of the findings. Fourthly, the lack of diversity in participant backgrounds, with many studies recruiting predominantly white and higher-income populations, restricts the applicability of the findings to broader, more diverse populations (36). This may introduce selection bias and limit the external validity of the study results. Future research should actively recruit more diverse participant groups, including different racial/ethnic groups, socioeconomic statuses, and geographic regions, to ensure the findings are representative of the general population. Fifthly, blinding issues in some studies pose a threat to the internal validity of the results. In several trials, neither the participants nor the outcome assessors were adequately blinded to the intervention allocation (37), which could have introduced performance and detection biases. Future studies should implement more rigorous blinding procedures, such as participant and assessor blinding, to minimize the risk of bias and enhance the credibility of the findings. Additionally, the search strategy may have missed some relevant gray literature despite efforts to include it. This could potentially lead to publication bias, as unpublished or difficult-to-access studies might have different results from those included in the analysis (38). Future reviews should employ more comprehensive gray literature search strategies, including contacting study authors directly and searching additional gray literature databases, to ensure a more complete representation of the existing evidence. Moreover, the absence of a pre-registered protocol for this review introduces potential bias and does not fully align with Cochrane’s guidance. This may affect the transparency and reproducibility of the review process. Future reviews should pre-register protocols on platforms such as PROSPERO, specifying eligibility criteria, search strategies, and analysis plans in advance to enhance transparency and reduce the risk of bias. Finally, the majority of studies included in this meta-analysis did not report on adverse events associated with the interventions. This limits our understanding of the safety profiles of IHT and IF in obese populations. Future research should systematically report on adverse events to provide a more comprehensive assessment of the risk–benefit balance of these interventions. Collectively, these limitations necessitate cautious interpretation of the findings while highlighting critical areas for methodological improvement in future studies.

### Combining IHT and IF

The overall interactions of IHT and IF in the meta-analysis sample imply that, where feasible, the implementation of the two together could offer enhanced metabolic and cognitive outcomes. It was suggested that clinicians develop a gradual approach to incorporating IHT and IF together, as synchronizing the two could become overwhelming for the patient. A gradual means to approach the plan could be that one starts with IF first to enable one to acclimatize to the concept of fasting in addition to the IHT practices. Based on clinical experience, we recommend allowing 4 weeks of IF adaptation before introducing IHT sessions, beginning with shorter durations (20–30 min) at moderate hypoxia levels (14–15% O₂) twice weekly, progressively increasing to protocol targets. This is also good to note that when both these two interventions are used, there is likely to be more supervision and monitoring needed, especially for patients with complications. For optimal outcomes and the best response to the treatment, the patient needs to be followed up at a later date with recommended assessments at 2-week intervals during the initial combination phase.

### Future directions

Although this meta-analysis supports IHT and IF as promising non-pharmacological interventions for obesity-related complications, key knowledge gaps require resolution before clinical implementation. Future studies must prioritize population diversity, as current cohorts predominantly represent high-income white populations despite metabolic conditions affecting 25% of US adults (39). Recruiting ethnically and socioeconomically diverse participants will enhance generalizability and identify beneficiary subgroups. Additionally, long-term trials (>12 months) are essential to evaluate intervention sustainability, given current studies’ limited durations (4–12 weeks) (40). Such research should determine whether metabolic/cognitive improvements persist and monitor potential late-onset adverse effects. Critically, our findings demonstrate IHT and IF significantly improve metabolic profiles—enhancing insulin sensitivity, reducing body weight, and optimizing lipid parameters—which collectively mitigate cardiovascular and diabetic risks (41, 42). Clinically, IF shows particular promise for insulin-resistant patients by improving glucose homeostasis (43), while IHT enhances mitochondrial efficiency in metabolically compromised individuals (44). The synergistic effect of combined IHT + IF offers superior outcomes for weight loss and cognitive function (45, 46), suggesting preventive potential against neurodegenerative processes (47). These interventions also ameliorate obesity-associated cognitive decline through reduced oxidative stress and neuroinflammation (48). Given their cost-effectiveness and low adverse event profile, IHT and IF should be integrated as adjuncts to conventional therapies, especially where pharmacological options are contraindicated or inaccessible (49). Priority research areas should include: (1) Mechanistic investigations into organokine regulation (particularly BDNF and leptin pathways); (2) Development and validation of pediatric-specific protocols; (3) Cost-effectiveness analyses in real-world healthcare settings; and (4) Standardized safety monitoring frameworks for adverse event reporting (69, 70).

## Conclusion

This meta-analysis demonstrates that IHT and IF, both individually and in combination, hold considerable promise as non-pharmacological interventions for combating obesity and its associated metabolic and cognitive complications. The substantial weight loss (mean reduction 6.3 kg), improved insulin sensitivity, enhanced lipid profiles, and cognitive benefits observed in the studies suggest that these interventions can provide meaningful clinical improvements. However, the limitations identified, including short intervention durations, heterogeneity in study populations and protocols, and underreporting of adverse events, must be addressed in future research. Long-term studies with diverse populations, standardized protocols, and rigorous methodologies are essential to confirm the sustained effectiveness and safety of IHT and IF. Despite these limitations, the current findings indicate that IHT and IF can be effective tools in the management of obesity, offering a cost-effective and accessible approach that can be integrated into holistic treatment plans. Specifically, the demonstrated efficacy supports implementing these interventions through a stepped protocol beginning with Intermittent Fasting and gradually incorporating hypoxia training, with appropriate clinical supervision. Future research should focus on elucidating the organokine-mediated mechanisms underlying cognitive improvements, optimizing protocols for special populations, and establishing long-term safety profiles to facilitate translation into routine clinical practice. Further exploration of these interventions in well-designed trials will be crucial to establish their optimal implementation and full potential in clinical practice.

## Data Availability

The original contributions presented in the study are included in the article/supplementary material, further inquiries can be directed to the corresponding author.
